# Sexual selection and cancer biology

**DOI:** 10.18632/oncotarget.4592

**Published:** 2015-06-23

**Authors:** Joshua B. Rubin

**Affiliations:** Departments of Pediatrics and Anatomy and Neurobiology, Washington University School of Medicine, St Louis, MO, USA

Darwin suggested that when males and females “differ in structure, colour or ornament” it is the result of sexual selection (“On the origin of species” by Charles Darwin). It has become increasingly apparent that in addition to overt sexual dimorphisms, there are substantial differences in underlying male and female biology that have relevance to human health and disease. It was recently reported that male astrocytes exhibit markedly greater tumorigenic responses to combined loss of neurofibromin and p53 function and activation of the EGF receptor pathway than do female astrocytes [1]. Moreover, differential RB regulation was shown to be essential for the manifestation of sex differences in tumorigenicity. It was suggested that cell intrinsic dissimilarities in tumor suppressor and oncogene effects provided novel insight into the basis for disparities that exist in glioblastoma rates and outcomes. It is likely however, that these findings are more broadly relevant to the overall greater rate of cancer diagnoses and cancer deaths in males. The experimental paradigm that was used for measuring sex-specific effects provides new opportunities and possibly, new requirements for basic and translational research. What makes this position so compelling is the ways in which sex differences in tumorigenicity can be related to fundamental sexual dimorphisms in growth, the vigorous evolutionary forces that created them, and the multiple developmental mechanisms that execute sex-specific growth strategies [2].

As any parent knows normative growth for boys and girls is substantially different. Not only do males typically grow to a greater size than females, but they also grow for a longer period of time. Sex related size differences that result in larger males are pervasive throughout the animal kingdom with few exceptions. Greater male size is a manifestation of greater male growth rates from the pre-implantation stage of embryogenesis to adulthood, with the brief period of exception during early puberty. The important questions are: Why are there differences in growth between males and females? What are the mechanistic foundations of sexual dimorphism? What are the implications of sexual dimorphism for the risks and manifestations of disorders in growth regulation such as cancer? What are the opportunities and requirements for studying sex differences in lab-based and clinical investigations in oncology?

Darwin recognized that sex differences were evolutionarily determined and he promulgated a theory of sexual selection, a distinct evolutionary process for shaping phenotypic differences within a species through competition between individuals of the same sex for reproductive success. Markedly different reproductive pressures on males and females have produced colorfully distinct strategies for success. Male reproductive success is limited by access to mates and consequently their reproductive strategy involves body size and displays that enhance their attractiveness to females and their ability to vanquish other males. This has been referred to as the “live fast, die young” blueprint for success [3]. Resources, not mates, limit female reproductive success. Consequently large body size and ostentatious displays are counterproductive. Instead, balancing growth with viability, particularly from gestation through weaning stages of offspring development is paramount for female reproductive success. Thus, there are differing growth optima for males and females and genetic conflict in growth regulatory pathways [4].

It is appealing to think about the relationship between differences in body size, the numbers of cells required and cancer risk as a simple equation in which bigger size and more cells results in greater cancer risk. However, the normal distribution of size between males and females overlaps significantly and is not likely to be the primary determinant of differences in cancer risk. Instead, the importance of sex-specific growth to cancer biology is more likely to lie in the homeostatic mechanisms that are in place to defend and preserve the differing optima. Normal distribution in size is a balance between male-derived influences that favor being larger (e.g. paternal imprinting of the *H19* locus) and female-derived influences that favor being smaller (e.g. maternal imprinting of the *IGF2* locus). The critical question may be, what happens when perturbations in growth regulation occur that shift normal growth towards greater or smaller growth rates and size. If loss of growth control were to move growth closer to the male optima but further from the female optima would the counter-regulatory response be the same, or would this be “allowed” in the male cell but “disallowed” in the female cell? This may be the key to the relationship between differences in growth and cancer at the earliest stages of cancer initiation when loss of tumor suppressor function or gain of oncogene function first alters growth. This could be the basis for the differential effects of combined loss of neurofibromin and p53 function.

Developmental consequences of in utero stressors provide important insight into these issues. Multiple studies of the impact that maternal nutrient deprivation and other in utero stressors have on fetal development reveal sex differences in viability and body size at birth [5]. Normal growth rates but increased fetal demise is more commonly observed for males while greater viability but decreased body size is more commonly observed for females. Underlying these differences are epigenetic mechanisms that preserve metabolism and growth stimulatory pathways in males and differing epigenetic mechanisms that slow growth, reduce nutrient requirements and increase immune activity in females [6]. A related set of observations suggests that epigenetic sex differences like these may be prevalent. The bed nucleus of the stria terminalis (BNST) is a highly sexually dimorphic region of the brain. An analysis of histone bivalency in male and female BNST revealed H3K4/K27 trimethylation at developmental and growth regulatory genes in males but in synaptic function genes in females [7]. These data may indicate that in response to perturbations in growth, males are poised to maintain their size, while females are poised to preserve their function.

With such strong evolutionarily designed and developmentally ensconced mechanisms, sexual dimorphism in growth, metabolism and immune function are bound to determine cancer risk and therapeutic responses. The importance of sex differences as a variable in research design should not be ignored. These findings should prompt investigators to examine oncogenic mechanisms separately in male and female experimental systems and to anticipate that therapeutics targeting growth regulatory pathways may have differing effects in male and female cancer patients.
